# Effect of different bleaching protocols on the color and translucency parameters of a new monochromatic universal composite resin

**DOI:** 10.34172/joddd.41450

**Published:** 2024-12-14

**Authors:** Mohammad Esmaeel Ebrahimi Chaharom, Mahmoud Bahari, Soodabeh Kimyai, Helia Bagban Nikzad

**Affiliations:** ^1^Department of Operative Dentistry, Faculty of Dentistry, Tabriz University of Medical Sciences, Tabriz, Iran; ^2^Dental and Periodontal Research Center, Tabriz University of Medical Sciences, Tabriz, Iran

**Keywords:** Bleaching agents, Color, Color perception, Composite resin, Laser, Peroxides

## Abstract

**Background.:**

Considering the few studies on the effect of bleaching protocols on the color properties of a newly developed monochromatic universal composite resin, the present study evaluated the effect of different bleaching protocols on the color change and translucency of Omnichroma (OMN) composite resin.

**Methods.:**

In this laboratory study, 45 cylindrical OMN composite resin disks with a diameter of 10 mm and a thickness of 2 mm were used. The samples were randomly divided into three groups (n=15) based on the bleaching protocols: group 1: 40% hydrogen peroxide (HP) for 20 minutes, twice a day for three sessions one week apart; group 2: 20% carbamide peroxide (CP) for 8 hours a day, for 14 consecutive days; group 3: 40% HP with Nd:YAG laser (HP+Nd:YAG laser) applied on the gel for 30 seconds twice. Baseline and post-bleaching measurements of color parameters were done using a Vita Easyshade spectrophotometer. Data was analyzed using paired-samples t-test, one-way ANOVA, and post hoc Tukey tests (*P*<0.05).

**Results.:**

All the bleaching protocols significantly changed the color (*P*<0.001) and translucency (*P*<0.001) parameters. CP caused the most color changes (*P*<0.05) and translucency (*P*<0.05), which were significant, and color and translucency changes caused by HP and HP+Nd:YAG laser were not significant (*P*>0.05).

**Conclusion.:**

Bleaching caused a noticeable change in the color and translucency of the OMN composite resin. The effect of the at-home bleaching protocol was greater than the in-office ones.

## Introduction

 To achieve imperceptible restorations, the restorative material must match the color and translucency of natural teeth. Commonly, color-matching techniques such as layering methods using composite resin materials with different shades that are adjusted with pigments are used to match the color of the restoration with the adjacent dental structures. For this reason, color matching in direct composite resin restorations is difficult because these techniques depend on the dentists’ experience level.^1^

 Recently, a new type of universal composite resin (Omnichroma; OMN, Tokuyama Dental, Tokyo, Japan) has been developed, in which the manufacturer claims that it is monochromatic and its structural color mechanism can mimic the color of the surrounding teeth regardless of its shade. OMN has no pigment, and its better performance in reproducing the color of different dental shades is due to its potential for better blending effect with tooth structures. The color appearance of a composite resin restoration is influenced by translucency and light transmission characteristics (straight linear transmission and light emission) and its colors (brightness, chroma, and hue).^2^ The size and shape of the filler particles are important factors in achieving the structural color phenomenon because the ambient light passes through the composite resin material. OMN is a nanohybrid composite resin with 260-nm spherical fillers with more linear light transmission properties without emitting light. The manufacturer claims that the structural color phenomenon occurs due to the fillers being of the same shape and size. According to the manufacturer, the composition of OMN is a mix of a uniformly sized supra-nanospherical filler of silicon dioxide (SiO_2_) and zirconium dioxide (ZrO_2_) with a particle size of 260 nm plus round-shaped composite resin fillers with the same characteristics. Furthermore, the monomer has a refractive index of 1.47 and 1.52 before and after polymerization, respectively, which means that the material’s transparency increases after polymerization.^3^

 Since its introduction by Haywood and Haymann in 1989, the bleaching method has become one of the most popular cosmetic treatments dentists offer. Materials used in bleaching include a variety of peroxides in gel or liquid form. The mechanism of action of these substances is generally based on the release of free radicals. In this way, unstable free radicals enter into a chemical reaction with colored molecules and make them smaller and lighter.^4,5^ Sometimes, light sources are used to increase the free radicals of whitening agents in this process. The main purpose of using the light source is not to whiten the teeth directly but to activate the whitening agent.^6,7^ Sağlam et al^8^ demonstrated that the use of laser, especially the Nd:YAG laser, could increase the efficiency of intracoronal bleaching with sodium perborate. Furthermore, Domínguez et al^9^ showed that the 532-nm wavelength of the Nd:YAG laser is suitable for increasing the bleaching effect.

 Regardless of the method and material used for bleaching, the restorations in the patient’s mouth may also be affected by bleaching. Bleaching material can cause changes in the structure of composite resins, such as increasing porosity surface roughness and reducing the hardness of composite resins. Furthermore, the possibility of bacterial adhesion and stain formation increases after the bleaching process.^7,10,11^ Several studies reported significant changes in the color and physical properties of microfilled and hybrid composite resins following the use of 6%‒15% carbamide peroxide (CP) and 30%‒35% hydrogen peroxide (HP).^4,12-14^ However, some studies showed no significant color change after vital bleaching with CP.^15-17^

 Therefore, dentists should note that composite resin restorations may not act in the same way that natural teeth respond to bleaching. If the restorative material has an excellent color match with the adjacent teeth before bleaching, this may not be the case after bleaching. Therefore, the final result after bleaching may be an esthetic failure. Patients should be aware that existing restorations may not match natural teeth after bleaching, and replacement may be needed for esthetic reasons.^4,18^

 Considering the few studies on the effect of bleaching strategies on the color properties of monochromatic universal composite resins, the purpose of this study was to evaluate the effect of different bleaching methods on the color change and translucency of a Omnichroma (OMN) universal composite resin.

## Methods

###  Sample preparation

 In this laboratory study, 45 cylindrical composite resin disks made of OMN composite resin (Tokuyama, Tokyo, Japan) with a diameter of 10 mm and a thickness of 2 mm were used. To cure the composite resin samples, the Elipar^TM^ S10 LED Curing Light light-curing device (3M ESPE, St. Paul MN, USA) was used with a constant light intensity of 1000 mW/cm^2^ and a curing time of 20 seconds according to the manufacturer’s instructions at a distance of 1 mm. Curing was done on the top and bottom of the samples, and before curing the final layer, a celluloid sheet and glass slab were used to make the surface smooth. Then, the samples were placed in distilled water for 24 hours at 37 °C. Surface polishing was done by 600-, 800-, and 1200-grit silicon carbide discs and Tor VM discs (Tor VM, Moscow, Russia). Finally, the samples were washed for 2 minutes in a JE27000 ultrasonic device (Juya Electronic, Tehran, Iran) with a frequency of 50-60 Hz and water pressure of 25‒60 Pascal and dried with airflow.^19^ Then, the samples were randomly divided into three groups of 15 according to the bleaching method.

###  Bleaching protocols

 In group 1, 40% HP (Opalescenceو Ultradent Products Inc., South Jordan, Utah, USA) was used for 20 minutes twice a day for 3 consecutive sessions one week apart.^7^ In group 2, the samples were covered with 20% CP whitening gel (Opalescence, Ultradent products. Inc., South Jordan, Utah, USA) for 8 hours a day for 14 consecutive days.^7^ After each session, the samples were washed with water for one minute and kept in distilled water at 25 ºC in a standard incubator.^7^ In group 3, the samples were covered with 40% HP whitening gel (Opalescence, Ultradent products. Inc., South Jordan, Utah, USA) with a gel thickness of 1.5 mm. Then, Nd:YAG laser (FIDELIS, Fotona, Slovenia) (HP + Nd:YAG laser) was applied to the gel for 30 seconds (2.5 W, 25 Hz, 320-μm fiber, at a 6-mm distance from the surface). After 60 seconds, the process was repeated. The whitening agent was washed off with distilled water 3 minutes after laser application.^20^

###  Color measurements

 To compare the color change of the samples, baseline evaluation (before bleaching) and evaluation after bleaching were undertaken. For this purpose, a spectrophotometer, Vita Easyshade (Zahnfabrik, Bad Säckingen, Germany), was used after calibration and using the CIELab system, in which L* indicates the amount of gray and determines the value or brightness, a* indicates the degree of inclination towards the red-green axis, and b* indicates the degree of inclination towards the blue-yellow axis. These values were measured three times for each sample, and finally, the average value was recorded.

 Then, the values before and after bleaching were compared, and color changes were calculated using the ΔE formula: ΔE^∗^ = [(ΔL^∗^)^2^ + (Δa^∗^)^2^ + (Δb^∗^)^2^]^1/2^. ΔL*, Δa*, and Δb* are the changes in L*, and a*, and b* parameters after bleaching protocols.^21^

###  Translucency measurements 

 To compare the changes in translucency of the samples, the L*, a*, and b* coordinates of each sample were determined using the same spectrophotometer before and after bleaching protocols. Translucency was evaluated by the translucency parameter (TP). The measurement was done three times for each sample, and the average was recorded. The procedure was such that the samples were evaluated for color parameters, once on a white background and again on a black background. TP was obtained by measuring the color difference of the samples between the two states of black background and white background according to the following formula:

 TP = [(L*_W_ – L*_B_)^2^ + (a*_W_ – a*_B_)^2^ + (b*_W_ – b*_B_)^2^]^1/2^

 where the subscript W indicates the color coordinates over the white background and the subscript B indicates those over the black background.^22^

###  Statistical analysis

 A paired samples t-test was used to compare translucency and color change before and after bleaching. One-way ANOVA was used to compare the average color change and translucency between different bleaching protocols. Post hoc Tukey tests were used to compare the two groups. The significance level in all the tests was considered < 5%. SPSS 21 was used for data analysis.

## Results

 Color parameters of OMN universal composite resin before and after bleaching are summarized in Table 1. The normality of the data was confirmed using the Kolmogorov-Smirnov test (*P* > 0.05).

**Table 1:** Descriptive statistics and comparison of color parameters before and after bleaching with three different methods

**Bleaching strategies**	**Color parameters**	**N**	**Before**		**After**		***P*** value^*^
			**Mean**	**SD**	**Mean**	**SD**	
Carbamide peroxide	l	15	75.960	2.886	81.940	2.960	< 0.001
	a	15	4.953	0.576	0.127	0.187	< 0.001
	b	15	16.000	2.212	12.567	1.661	< 0.001
Hydrogen peroxide	l	15	76.933	1.177	83.327	3.161	< 0.001
	a	15	1.487	0.488	-0.380	0.161	< 0.001
	b	15	17.187	3.131	14.047	1.012	< 0.001
Nd:YAG laser	l	15	78.327	2.405	84.033	1.153	< 0.001
	a	15	1.473	0.559	-0.347	0.151	< 0.001
	b	15	16.180	1.133	12.307	0.915	< 0.001

^*^ Paired-samples t-test.

###  Bleaching protocols

 The paired samples t-test showed that all the three bleaching protocols significantly changed the color and translucency parameters of the OMN (*P* < 0.001) (Table 1).

###  Color changes

**Figure 1:**
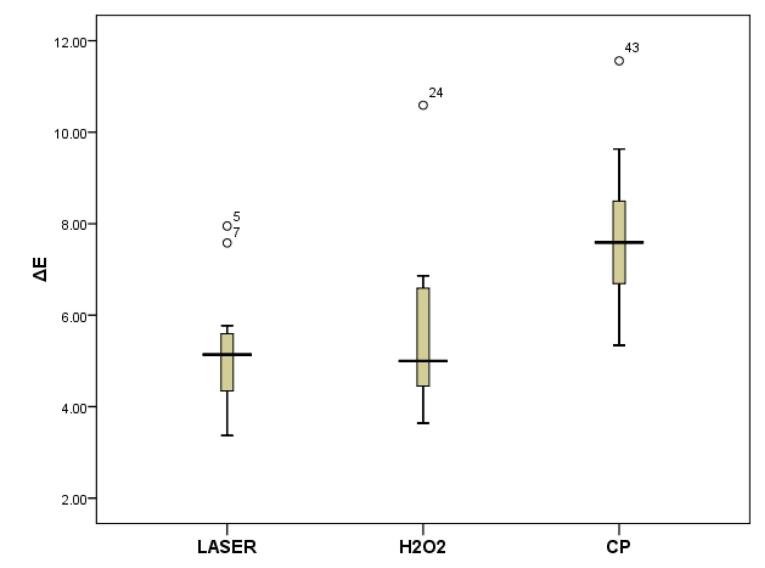
Comparison of color changes (ΔE) in three bleaching protocols

 One-way ANOVA showed a significant difference in the color changes (ΔE) of OMN between the three bleaching methods (*P* < 0.001). Post hoc Tukey tests showed that CP caused the greatest translucency change, which was significantly more than those with HP (*P* = 0.002) and HP + Nd:YAG laser (*P* < 0.001), and the color changes caused by HP and HP + Nd:YAG laser were not significantly different (*P* > 0.05) (Figure 1).

###  Translucency parameter

**Figure 2:**
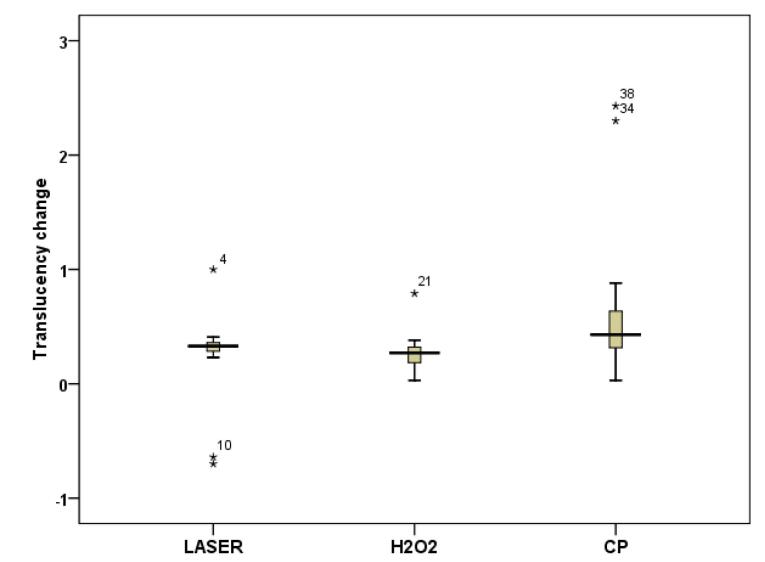
Comparison of translucency changes in three bleaching protocols

 Regarding translucency, one-way ANOVA showed a significant difference in translucency changes between the three bleaching methods (*P* = 0.03). Post hoc Tukey tests showed that CP significantly caused the greatest translucency changes that were significantly more than HP (*P* = 0.03) and HP + Nd:YAG laser (*P* = 0.02) groups, and the translucency changes caused by HP and HP + Nd:YAG laser were not significantly different (*P* > 0.05) (Figure 2).

## Discussion

 During bleaching, especially at-home bleaching, existing restorations are affected by bleaching materials for a long time. It has been shown that bleaching agents may cause discoloration in the restoration that the patients perceive and consider unacceptable.^4,23,24^ Therefore, this study investigated the effects of different bleaching protocols on the color changes and translucency of OMN universal composite resin.

 Different methods have been proposed to measure the color changes of teeth after bleaching. It includes the subjective method with visual analysis related to the standard color guide and the objective method, which includes the spectrophotometer, colorimeter, and analysis software. Eye methods are not accurate enough. Spectrophotometry has been introduced as a repeatable and accurate method of color measurement. In this study, a spectrophotometer and CIELab system were used to measure color changes; the CIELab system is known for its international validity, ease of use, and high reliability. The CIELab color space defines each color through three values ​​and places each color at a specific point on a sphere. The vertical color axis L* is related to the object’s lightness and has a value from 0 for complete black to 100 for complete white. In the a* coordinate, the positive value indicates red, and the negative value indicates green. In the b* coordinate, the positive b* value indicates yellow, and blue is the negative b* value. All the measurements were performed on a white background to avoid any absorption of color parameters. The values ​​measured at the beginning and at the end of the experiment were put in the ΔE formula. Changes in all the three color parameters (∆L*, ∆a*, ∆b*) were obtained. When ΔL*, ∆a*, and ∆b* are included in the formula, the ∆E value is obtained, i.e., the difference in the position of two points in the three-dimensional CIEL*a*b* color space, which represents the total color change (∆E) that occurred between the two measurements. ΔE between 0 and 3.2 indicates that although a color change has occurred, it is not visually perceptible and may be clinically acceptable. ΔE* > 1 can be understood in visual evaluation (unaided eye) in half of the people, and ΔE > 3.3 is clinically unacceptable.^4,12,13,25^

 According to the results of this study, all the three bleaching protocols, including HP, CP, and HP + Nd:YAG laser, caused a significant difference in the color parameters of OMN. In all the three bleaching protocols, the increase in L* value indicates increases in values. a* showed a green shift (negative values) in HP + Nd:YAG laser and HP and a red shift (positive value) in CP. Concerning the b* parameter, bleaching decreased in all the three protocols and showed a decrease in yellowness.

 Similarly, Kurtulmus-Yilmaz et al^24^ compared the effect of home-bleaching application of 10% CP and 10% HP on the color changes of five composite resins (Reflexions, Grandio, Gradia Direct, Clearfil Majesty Esthetic, and Ceram-X Mono) and demonstrated that application of CP and HP resulted in clinically unacceptable color changes (ΔE > 3.3) for all the composite resins. There were significant color differences between the control group and bleached specimens. However, no significant color difference was found between CP and HP. In comparing the results of Kurtulmus-Yilmaz and colleagues’ study with the current study, it should be noted that the bleaching protocol in the study above, regardless of the difference in the materials used for bleaching, was performed with an at-home protocol with the same duration of bleaching materials’ application. However, in the current study, the protocols and duration of applying bleaching materials were different, and the amount of color change observed in the at-home protocol was significantly higher than the other two protocols, both of which are based on the in-office protocol because of longer application time of bleaching agents in the at-home protocol. In addition, Hubbezoglu et al^17^ demonstrated that color changes were greater in at-home bleaching protocol with 16% CP and 35% HP than in-office bleaching protocol with CP 37%. So, it can be concluded that bleaching protocol and application time are of greater importance than the percentage and type of bleaching material.

 Öztürk et al^23^ reported that the effect of different bleaching protocols, including 35% HP and 20% CP, on the color change of Estelite Posterior composite resin was beyond the clinically acceptable limit. However, ΔE values for the resin matrix ceramics (ormocer-based) after the bleaching procedures were below the clinically acceptable values, regardless of the bleaching agent. So, they concluded that the bleaching protocol and composite resin type were more important than the bleaching agents’ composition and percentage. In this regard, the results were also consistent with Irawan et al,^4^ who showed that the color change with 20% CP using at-home was more than 40% CP in the in-office procedure. Furthermore, similar to the present study, ∆Es > 6 have also been reported by other studies that tested in-office bleaching on tooth samples.^26,27^

 In general, the mechanism of composite resin color change during bleaching includes oxidation of surface pigments, oxidation of amine compounds, which are responsible for color stability over time, and failure of resin matrix that is poorly polymerized. These effects can be intensified by using light sources during bleaching. Hydrogen peroxide is an aggressive oxidizing agent that can break the organic matrix into free radicals. These free radicals eventually combine to form molecular oxygen and water. Parts of this chemical reaction may accelerate the hydrolytic decomposition of the composite resin, causing its color to change. Furthermore, the organic matrix of composite resin is susceptible to chemical changes caused by bleaching agents. Therefore, a composite resin with more resin is more prone to decomposition and color change. The free radicals created by peroxides affect the filler‒resin interface and cause filler‒matrix debonding. Microscopic cracks are created, increasing surface roughness and spreading the bleaching agent.^14,17^

 In contrast to this study, where the bleaching effect was examined on composite discs, AlHabdan et al^25^ examined the effect of bleaching on the color change of OMN on natural teeth in Class V cavities and demonstrated that ΔE values of OMN before and after bleaching were 3.529 and before bleaching and two weeks post-bleaching were 3.651 with no statistically significant difference. These values are higher than the perceivable threshold of 3.3, which shows a significant color change after bleaching, and this color change was stable after 2 weeks of bleaching. This is also consistent with Evans,^28^ who showed significant color changes in OMN after bleaching. Positive ΔL* values indicate that the specimens became lighter in color after bleaching. So, bleaching could bleach OMN specimens in a manner similar to that of tooth structures. In addition, Mohamed et al^29^ evaluated the evidence of shade matching and color change of OMN composite resin class V restorations on 10 extracted teeth with varying shades (A1-D4) over a month after bleaching. The average color change after bleaching was 3 shades. Shade matching scores by the clinicians showed that the composite resin matched 100% for all teeth at different time intervals. There was no significant difference in the L*, a*, and b* parameters between the restoration and tooth at each time interval. Furthermore, in a more recent study, Forabosco et al^30^ evaluated the color match of different single-shade class V composite resin restorations (Omnichroma, Clearfil Majesty ES-2 Universal, Essentia Universal, and Venus Diamond One) with surrounding tooth structure before and after bleaching. Spectrophotometric evaluations revealed statistically significant differences between materials in some time points. However, the visual analysis showed excellent and very good grades of color matching, irrespective of the materials and time points. Finally, considering and comparing the findings of the present study and other similar studies in this regard, it can be concluded that OMN can shift its color after bleaching in a manner similar to that of natural tooth structures to achieve a good color match with the surrounding tooth structure, before and after professional bleaching.

 Translucency is the ability of a material to show its background, and it can change from partial translucency to a state between full transparency and complete translucency. Translucency is a basic criterion for esthetics, and in terms of importance in obtaining restorations with natural appearance, it is comparable to the color parameters.^23,24^ The background color of the tooth in the oral cavity can affect the perceived color of the composite resin restoration, which depends on the translucency of the composite resin.^2^ It has been shown that bleaching agents increase the roughness of the composite resin surface, and as the surface roughness increases, the translucency decreases.^24^ The effect of bleaching agents on the surface roughness of composite resin can be related to its concentration. Some studies have shown an increase in surface roughness after bleaching. It is claimed that these changes are due to the chemical decomposition of the resin matrix depending on the concentration or repeated application of peroxide.^10,11^

 In the present study, all the three types of bleaching protocols caused significant differences in translucency. CP showed the most significant changes, and HP had no significant difference from HP + laser. Contrary to the results of this study, according to Öztürk et al,^23^ no clinically perceptible change in the translucency of Estelite Posterior composite resin was observed before and after bleaching. Furthermore, Kurtulmus-Yilmaz et al^24^ demonstrated that nanocomposites, nanohybrids with an ormocer base, and microhybrids did not show significant changes in terms of TP after bleaching with 10% CP and 10% HP.

 The discrepancy between the results of various studies might be due to different bleaching protocols, different concentrations of bleaching agents, different microstructures of composite materials, different light sources, curing methods, colorimetric methods, and background colors during color measurement. Considering the limitations of the current study, monochromatic universal composite resins need more studies regarding the relationship between bleaching, color change, surface roughness, and the effect of intraoral conditions and aging.

## Conclusion

 Different bleaching protocols can cause a noticeable shift in the color and translucency parameter of the OMN monochromatic universal composite resin. The effectiveness of the at-home bleaching was greater than the in-office protocols, probably due to the longer contact time of the bleaching agent during the at-home procedure. Comparing the findings of the present study with other studies can lead to the conclusion that the color of OMN shifts after bleaching similarly to that of natural tooth structures and can achieve a good color match with the surrounding tooth structure after professional bleaching.

## Acknowledgments

 The authors extend their appreciation to the Vice Chancellor for Research and Technology, Tabriz University of Medical Sciences, for the financial support of this research.

## Competing Interests

 The authors declare no competing interests with regard to the authorship and/or publication of this article.

## Ethical Approval

 The study was approved by the ethical committee of Tabriz University of Medical Sciences, Tabriz, Iran (Ethics No. IR.TBZMED.VCR.REC.1402.028).
